# Perceived enablers of exclusive breastfeeding by teenage mothers in Ghana

**DOI:** 10.4102/safp.v62i1.5108

**Published:** 2020-09-21

**Authors:** Angela K. Acheampong, Makombo Ganga-Limando, Lydia Aziato

**Affiliations:** 1 School of Nursing, Wisconsin International University College, Accra, Ghana; 2 Department of Health Studies, University of South Africa, Pretoria, South Africa; 3 Department of Adult Health, School of Nursing, University of Ghana, Legon, Accra, Ghana

**Keywords:** breastfeeding, exclusive breastfeeding enablers, qualitative approach, teenage mothers, breastfeeding education

## Abstract

**Background:**

Exclusive breastfeeding for the first 6 months can prevent diseases, boost immunity and improve quality of lives of infants. Ghana implemented programmes aimed at reaching the global target of increasing exclusive breastfeeding for the first 6 months to at least 50% by the year 2025. The country witnessed a decline in the overall rate of exclusive breastfeeding and an increase in the number of teenage mothers. Globally, teenage mothers are less likely to breastfeed than mothers of other age groups. Understanding enablers of exclusive breastfeeding by teenage mothers is important for any intervention aimed at improving exclusive breastfeeding rates and the quality of lives of infants.

**Method:**

The study used a qualitative, exploratory, descriptive and contextual design, with focus groups. A total of six group discussions were conducted with 30 pregnant teenagers recruited from six public hospitals.

**Results:**

Seven enablers emerged from the analysis of data. These included positive beliefs about the benefits of breast milk, family history of positive exclusive breastfeeding outcomes, support of the intimate partner, approval of closed-family members, expert opinions of antenatal care staff, teenage-oriented breastfeeding education and community-based breastfeeding education.

**Conclusion:**

Health professionals and policy makers could learn from these enablers and use them to promote exclusive breastfeeding practices amongst teenage mothers in Ghana.

## Introduction

Ghana subscribed to the global target of increasing exclusive breastfeeding for the first 6 months to at least 50% by the year 2025.^1^ Mothers are encouraged to give breast milk to their infants within the first hour after delivery and continue to exclusively breastfeed for at least 6 months.^2^ Breast milk has the advantage of being readily available, free of charge and contains protective agents such as phagocytes, lactoferrin, oligosaccharides and immunoglobulin which help protect against common childhood illnesses such as diarrhoea and respiratory infections.^2^ Exclusive breastfeeding has the single largest potential impact on child mortality compared to any preventive intervention. It reduces the rate of illnesses amongst children, improves the mental capabilities and immunity and also gives children a better chance of survival. It provides infants complete nutrition for healthy growth and provides protection against life-threatening diseases.^3^ If practiced between the ages of 0 and 23 months, it could reduce the death count by about 800 000 amongst children under 5 years of age.^2^ In spite of the commitment to the global target and the benefits of exclusive breastfeeding, the overall rates of exclusive breastfeeding (0–5 months) in Ghana declined from 63.7% in 2008 to 52.3% in 2014 with 4.5% of mothers following exclusive breastfeeding for at least 6 months.^4^ In Ghana, 16.2% become mothers by the age of 18 years; however breastfeeding data are not aggregated based on mothers age groups. Globally, studies showed that teenage mothers were less likely to practice exclusive breastfeeding as compared to their older counterparts and have a more rapid discontinuation rate.^5^ It is also known that most teenage mothers are unprepared for motherhood tasks including exclusive breastfeeding.^6^

It is argued that the decision to select the breastfeeding methods is often made by the mothers during the prenatal period. But this decision might be influenced by several other factors. Studies confirmed that personal positive beliefs about the benefits and value of breast milk, previous experiences with breastfeeding, positive intention towards breastfeeding, higher level of education, frequent antenatal visits, birth intervals and support from family members, especially spouses of mothers and supportive spouses can act as enablers of exclusive breastfeeding by mothers including teenage mothers.^7^ In addition, teenage mothers in Ghana are faced with socio-cultural challenges that might influence their decision to consider exclusive breastfeeding. They are often stigmatised and rejected by family members. Family members deem it shameful for an unmarried teenage girl to be impregnated.^8^ This study sought to understand factors that facilitate the teenage mothers’ decision to consider exclusive breastfeeding in such a social context. The purpose was to describe and explore the perceived enablers of exclusive breastfeeding by teenage mothers in the social context of Ghana.

## Research methodology

### Design

This study used a qualitative, exploratory, descriptive and contextual design, with focus groups (FGs) to depict the perceived enablers of exclusive breastfeeding by teenage mothers in Ghana.

### Setting

The study was conducted in six public hospitals in the greater Accra region of Ghana. These facilities serve a population of around 1 848 614.^4^

### Sample and sampling

The participants for this study were selected from the antenatal care services. Purposive sampling was performed to select pregnant teenagers aged 13–19 years, who attended at least three antenatal care visits and who were willing to participate in the FGs. The final sample comprised 30 pregnant teenagers who met the above criteria. All 30 participants were living with their parents; 20 received some form of education (ranging from primary to senior high levels), 21 were unemployed and 9 were self-employed.

### Data collection

Data collection was conducted over the period from October through December 2016. The researcher made six FGs with five participants in each group. The researcher grouped the participants according to their socio-economic background in order to stimulate discussions. The researcher conducted the discussions in one of the main local languages (Twi) spoken in Ghana. The researcher did not require an interpreter because of her proficiency in this language, and her understanding of the cultural idioms associated with this language. One key question that guided the discussions was: ‘what do you view as facilitators of exclusive breastfeeding by teenage mothers in the social context of Ghana?’. Discussions commenced with group rules and the presentation of the definition of exclusive breastfeeding.^2^ The researcher used interviewing techniques that ensured active and sustained discussions. Probing questions were used, when appropriate, to enrich data. Each group discussion lasted approximately 90 minutes. The discussions were digitally recorded and checked for quality.

### Data management and analysis

The researcher started by transcribing data verbatim from the local language within 24 hours. The transcribed data were immediately translated into English. Two experts in the Twi and English languages checked the translated data against the transcripts and digital records. Both of them were satisfied with the accuracy and contextual meaning of the translated data. The researcher used group-level thematic and content analyses.^9^ After assimilating the data, the researcher developed a coding scheme in which the concepts, sub-themes and themes were labelled and summarised. Finally, the sub-themes were compared in order to arrive at major themes. This process was managed by making use of NVivo software, version 11.

### Trustworthiness

The study ensured trustworthiness through the application of strategies described by Taylor. Member checking and the neutrality of the researcher during the discussions, and the triangulation of data, using independent coding and peer review were used to ensure the confidence in the truth of data generated and their interpretation. Data collection and analyses were carried out concurrently and this ensured that emerging themes were explored in subsequent discussions to bring out the full meanings of the themes. The researcher ensured consistency by phrasing questions in the same manner in all the FGs. The research team studied the themes to ensure that all the data were included in the study. Field notes assisted in the verification of findings. Verbatim quotes supported the findings.

### Ethical consideration

The study adhered to the universal ethical principles guiding the research with human subjects. Ethical approval to conduct the study was obtained from both the research and ethics committees of the University of South Africa (HSHDC/5-48/2016) and the Institutional Review Committee of Ghana Health Service (GHS-ERC: 07-11-2016).

## Results

Three themes with seven sub-themes emerged from the analysis of data. The seven sub-themes included positive beliefs about the benefits of breast milk, family history of positive exclusive breastfeeding outcomes, support of the intimate partner, approval of closed-family members, expert opinions of antenatal care staff, teenage-oriented breastfeeding education and community-based breastfeeding education. The three themes included attitude towards exclusive breastfeeding, social supports of significant others and holistic breastfeeding education.

### Theme 1: Attitude towards exclusive breastfeeding

This theme referred to the influence of the participants’ personal beliefs about the benefits of breast milk on the teenage mothers’ decision to exclusively breastfeed. This positive attitude was derived from positive beliefs about the benefits of breast milk and family history of positive exclusive breastfeeding outcomes.

#### Positive beliefs about the benefits of breast milk

The motivation to exclusively breastfeed was derived from the participants’ recognition of the nutritional value of breast milk for the infants. This motivation varied according to the level of beliefs about the benefits of breast milk. Participants who were fully convinced about the nutritional value of breast milk for the infants were certain about exclusively breastfeeding their infants. ‘I have decided to feed my baby with breast milk alone for six months because breast milk has everything that my baby needs to be strong, intelligent and healthy’. Participants who were not fully convinced about the nutritional value of breast milk preferred to adopt an attitude of ‘try and see’: ‘I would try exclusive breastfeeding after delivery to see if all the benefits they claim are really true. If not, I would definitely give additional food before six months’.

#### Family history of positive exclusive breastfeeding outcomes

The motivation to exclusively breastfeed emerged from the participants’ witnessing positive exclusive breastfeeding outcomes in the family:

‘I would definitely feed my baby with only breast milk without any additional water or food for six months because my mother did the same without any problem. I saw that it makes the baby grow with love and to bond with the mother.’ (FG 2, 13 years unemployed)

### Theme 2: Social supports of significant others

This theme referred to the supportive opinion held by people viewed by participants as having a great influence on the teenage mothers’ decision to exclusively breastfeed. Intimate partners, closed family members (mothers, grandmothers and sisters) and antenatal care staff emerged as significant others.

#### Support of the intimate partner

The intimate partners influenced the decision to exclusively breastfeed through their approval of breastfeeding and providing financial assistance. Participants attributed the approval of breastfeeding by intimate partners to the decision to initiate exclusive breastfeeding. They attributed financial assistance to the duration of exclusive breastfeeding:

‘I know that I have to follow what my partner wants me to do. He would like me to feed my baby with the breast milk only for the first six months. But he must also provide financial assistance to allow me to maintain exclusive breastfeeding for six months.’ (FG 6, 16 year unemployment)

#### Approval of closed-family members

Participants viewed the approvals of breastfeeding by closed-family members, that is mothers, grandmothers and sisters as strong motivators of exclusive breastfeeding. ‘My own decision about exclusive breastfeeding does not matter. I have to follow what my family members will tell me to do’. However, a mother’s approval was the most powerful motivator. It deprived the participants of the ability to take independent decision:

‘My mother’s words have the most influence on my decisions. My mother knows everything about delivery and babies. So if my mother says that I should breastfeed my baby for six months without additional water or food, I would definitely do it.’ (FG 3, 18 years self-employed)

#### Expert opinions of the antenatal care staff

The expert opinions of the antenatal care staff (nurses and midwives) emerged as motivators of exclusive breastfeeding. Participants believed that the expert advices of nurses were worth following:

‘If nurses tell me to feed my baby with breast milk without adding water or food for the first six months, I will do it because I trust their expertise as health professionals.’ (FG 4, 19 years -self-employed)

### Theme 3: Holistic breastfeeding education

Holistic breastfeeding education referred to an education geared towards the specific needs of teenage mothers and at promoting community support. Participants would consider exclusive breastfeeding if breastfeeding education addresses their specific needs and the social barriers to exclusive breastfeeding at the community level. Teenage-oriented breastfeeding education and community-based breastfeeding education emerged as sub-themes of holistic breastfeeding.

#### Teenage-oriented breastfeeding education

Participants believed that teenage-oriented breastfeeding education would increase their self-confidence. Such education should include amongst others practical demonstration on breastfeeding, maternal and infant nutritional actions, assertiveness and barriers to exclusive breastfeeding. Participants viewed self-confidence as reinforcing their motivation to exclusively breastfeed:

‘As for me, I am young and have never given birth before but they (nurses and midwives) spend a lot of time telling us about the benefits of breastfeeding. They do not consider the fact that we never breastfed before. They must demonstrate to us how to actually breastfeed our babies and take time to repeat it once we delivered. This will definitely motivate us to exclusively breastfeed our babies for six months without problems.’ (FG 1, 14 years unemployed)

#### Community-based breastfeeding education

Participants argued that community-based education would create an environment supportive to their decision for exclusive breastfeeding:

‘It is important that nurses come home and educate our relatives and community members about exclusive breastfeeding. It would assist in overcoming social barriers and support our decisions to exclusively breastfeed our babies for at least six months.’ (FG 3, 18 years self-employed)

## Discussion

The study provided an understanding of perceived enablers of exclusive breastfeeding by teenage mothers in Ghana. It emerged that positive attitude towards exclusive breastfeeding, social supports of significant others and holistic breastfeeding education motivate teenage mothers to consider exclusive breastfeeding. The Theory of Planned Behaviour supports the role of the attitude and social supports of significant others in influencing an individual’s behaviour. The theory argued that an individual’s attitude or overall estimate of and reaction to the outcome of a particular behaviour might lead to a positive attitude and consequently the actual performance of that behaviour. It further argued that an individual’s belief about whether significant others support the intended behaviour influences his or her performance related to that particular behaviour.^10^ Previous studies recognised the role of all the emerged sub-themes in enabling exclusive breastfeeding by teenage and mothers belonging to other age groups.

Studies conducted amongst teenage mothers in Canada,^11^ in Ecuador,^12^ in Hong Kong^13^ and in the United States^14^ identified positive beliefs about the benefits of breast milk, family history of positive exclusive breastfeeding outcomes, support of the intimate partner, approval of closed-family members and expert opinions of antenatal care staff as enablers of exclusive breastfeeding by teenage mothers. Contrary to the study conducted in Canada,^11^ where teenage mothers had ownership over their decision to exclusively breastfeed, teenage mothers in this study felt deprived of the ownership over their decision to exclusively breastfeed. Could the socio-cultural context explain this difference? Canadian society has a more liberal social environment that allows teenage to make independent decisions as compared to those in Ghana, who live in a society where compliance to social and cultural norms is a norm. This is reflected by the following statement of participants: ‘my own decision about exclusive breastfeeding does not matter. I have to follow what my family members will tell me to do’.

Randomised controlled trial studies of breastfeeding education conducted in Japan,^15^ in the United States^16^ and in Hong Kong^17^ as part of baby-friendly hospitals identified the need-based breastfeeding education and community-based breastfeeding education as enablers of the intention and actual exclusive breastfeeding by teenage mothers and those belonging to other age groups.

## Conclusion

The researcher was interested in understanding factors that facilitate the teenage mothers’ decision to consider exclusive breastfeeding in the social context of Ghana. The study suggested that exclusive breastfeeding by teenage mothers in the social context of Ghana is enabled by the personal attitudes of teenage mothers towards exclusive breastfeeding, social support of significant others and holistic breastfeeding education. It highlighted the importance of social norms in the decision-making process of exclusive breastfeeding by teenage mothers in Ghana. Therefore, any effort by health professionals and policy makers to promote exclusive breastfeeding by teenage mothers in Ghana must strengthen these factors.

## References

[1] World Health Organization, UNICEF. Global nutrition targets 2025: Breastfeeding policy brief (WHO/NMH/NHD/14.7) [homepage on the Internet]. [cited 2018 July 20]. Geneva: World Health Organization; c2014. Available from: https://www.who.int/nutrition/publications/globaltargets2025_policybrief_breastfeeding

[2] World Health Organization. Guideline: Protecting, promoting and supporting breastfeeding in facilities providing maternity and newborn services [homepage on the Internet]. Geneva: World Health Organization; c2017. Licence: CC BY-NC-SA 3.0 IGO. Available from: https://apps.who.int/iris 29565522

[3] Haile D, Biadgilign S. Higher breastfeeding performance index is associated with lower risk of illness in infants under six months in Ethiopia. Int Breastfeed J. 2015;10(1):32. 10.1186/s13006-015-0057-2 26617666 PMC4662817

[4] District Health Survey Ghana. Demographic and health survey of Ghana [homepage on the Internet]. Accra, Ghana: Demographic and Health Survey Programme, Greater Accra; 2016 [cited 2018 July 18]. Available from: https://www.dhsprogram.com

[5] Poole SN, Gephart SM. State of the science for practice to promote breastfeeding success among young mothers. Newborn Infant Nurs Rev. 2014;14(3):112–118. 10.1053/j.nainr.2014.06.009

[6] Doku D. Substance use and risky sexual behaviours among sexually experienced Ghanaian youth. BMC Public Health. 2012;12(1):571. 10.1186/1471-2458-12-571 22839700 PMC3517501

[7] Rollins NC, Bhandari N, Hajeebhoy N, et al. Why invest, and what it will take to improve breastfeeding practices? Lancet. 2016;387(10017):491–504. 10.1016/S0140-6736(15)01044-2 26869576

[8] Tampah-Naah AM, Kumi-Kyereme A. Determinants of exclusive breastfeeding among mothers in Ghana: A cross-sectional study. Int Breastfeed J. 2013;8(1):13. 10.1186/1746-4358-8-13 PMC3852847 24119727

[9] Padgett DK. Qualitative and mixed methods in public health. Thousand Oaks, CA: Sage; 2011.

[10] Ajzen I. The theory of planned behaviour: Reactions and reflections. Psychol Health. 2011;26(9):1113–1127. 10.1080/08870446.2011.613995 21929476

[11] Nestt SA, Campbell KA, Jack SM, Robinson H, Piehl K, Bogdan JC. Canadian adolescent mothers’ perceptions of influences on breastfeeding decisions: A qualitative descriptive study. BMC Pregnancy Childbirth. 2012;12:149. 10.1186/1471-2393-12-149 23234260 PMC3534235

[12] Jara-Placios MA, Cornejo AC, Pelaez GA, Verdesoto J, Galvis AA. Prevalence and determinants of exclusive breastfeeding among adolescent mothers from Quito, Ecuador: A cross-sectional study. Int Breastfeed J. 2015;10:33. 10.1186/s13006-015-0058-1 26692888 PMC4676122

[13] Lok KYW, Bai DL, Tarrant M. Predictors of breastfeeding initiation in Hong Kong and Mainland China born mothers. BMC Pregnancy Childbirth. 2015;15(1):286. 10.1186/s12884-015-0719-5 26531299 PMC4632339

[14] Smith PH, Coley SL, Labbok MH, Cupito S, Nwokah E. Early breastfeeding experiences of adolescent mothers: A qualitative prospective study. Int Breastfeed J. 2012;7(1):13. 10.1186/1746-4358-7-13 23020833 PMC3565878

[15] Otsuka K, Taguri M, Dennis CL, et al. Effectiveness of a breastfeeding self-efficacy intervention: Do hospital practices make a difference? Matern Child Health J. 2014;18(1):296–306. 10.1007/s10995-013-1265-2 23592322 PMC3880483

[16] Wambach KA, Aaronson L, Breedlove G, Domian EW, Rojjanasrirat W, Yeh H-W. A randomized controlled trial of breastfeeding support and education for adolescent mothers. West J Nurs Res. 2011;33(4):486–505. 10.1177/0193945910380408 20876551

[17] Tarrant M, Wu KM, Fong DY, et al. Impact of baby-friendly hospital practices on breastfeeding in Hong Kong. Birth. 2011;38(3):238–245. 10.1111/j.1523-536X.2011.00483.x 21884232

